# Aberrant Expression of Proteins Involved in Signal Transduction and DNA Repair Pathways in Lung Cancer and Their Association with Clinical Parameters

**DOI:** 10.1371/journal.pone.0031087

**Published:** 2012-02-10

**Authors:** Yong He, Zhen Zhou, Wayne L. Hofstetter, Yanbin Zhou, Wenxian Hu, Chengcheng Guo, Li Wang, Wei Guo, Apar Pataer, Arlene M. Correa, Yiling Lu, Jing Wang, Lixia Diao, Lauren Averett Byers, Ignacio I. Wistuba, Jack A. Roth, Stephen G. Swisher, John V. Heymach, Bingliang Fang

**Affiliations:** 1 Department of Thoracic Surgery, Daping Hospital, Third Military Medical University, Chongqing, China; 2 Shanghai Lung Tumor Clinic Medical Center, Shanghai Chest Hospital Affiliated to Shanghai Jiaotong University, Shanghai, China; 3 Department of Thoracic and Cardiovascular Surgery, University of Texas M. D. Anderson Cancer Center, Houston, Texas, United States of America; 4 Department of Systems Biology, University of Texas M. D. Anderson Cancer Center, Houston, Texas, United States of America; 5 Department of Bioinformatics and Computation Biology, University of Texas M. D. Anderson Cancer Center, Houston, Texas, United States of America; 6 Departments of Thoracic and Head and Neck Medical Oncology, University of Texas M. D. Anderson Cancer Center, Houston, Texas, United States of America; 7 Department of Pathology, University of Texas M. D. Anderson Cancer Center, Houston, Texas, United States of America; Virginia Commonwealth University, United States of America

## Abstract

**Background:**

Because cell signaling and cell metabolic pathways are executed through proteins, protein signatures in primary tumors are useful for identifying key nodes in signaling networks whose alteration is associated with malignancy and/or clinical outcomes. This study aimed to determine protein signatures in primary lung cancer tissues.

**Methodology/ Principal Findings:**

We analyzed 126 proteins and/or protein phosphorylation sites in case-matched normal and tumor samples from 101 lung cancer patients with reverse-phase protein array (RPPA) assay. The results showed that 18 molecules were significantly different (*p*<0.05) by at least 30% between normal and tumor tissues. Most of those molecules play roles in cell proliferation, DNA repair, signal transduction and lipid metabolism, or function as cell surface/matrix proteins. We also validated RPPA results by Western blot and/or immunohistochemical analyses for some of those molecules. Statistical analyses showed that Ku80 levels were significantly higher in tumors of nonsmokers than in those of smokers. Cyclin B1 levels were significantly overexpressed in poorly differentiated tumors while Cox2 levels were significantly overexpressed in neuroendocrinal tumors. A high level of Stat5 is associated with favorable survival outcome for patients treated with surgery.

**Conclusions/ Significance:**

Our results revealed that some molecules involved in DNA damage/repair, signal transductions, lipid metabolism, and cell proliferation were drastically aberrant in lung cancer tissues, and Stat5 may serve a molecular marker for prognosis of lung cancers.

## Introduction

Molecular profiling of lung cancer through gene array assays for mRNA and microRNA has led to the identification of molecular signatures that are potentially useful for predicting patient survival and disease relapse and/or response to individual chemotherapeutic drugs based on hierarchical and probabilistic clustering of mRNA [1] and microRNA levels [2]. Also, studies of single-nucleotide polymorphisms of genomic DNA have led to the identification of potential gene loci in the chromosome 15q25 region [3] that encode nicotinic acetylcholine receptor subunit genes that are highly associated with lung cancer susceptibility. Nevertheless, for most genes, there is no significant correlation between mRNA and protein levels [4]. Thus, the key signaling pathways that reflect the disease-transforming processes remain to be identified. Because most signal transduction and pathway regulation are conducted by proteins undergoing posttranscriptional modification, such as phosphorylation, which cannot be detected by DNA, mRNA, or miRNA analyses, characterization of protein levels and protein phosphorylation status is needed to obtain protein signatures that reflect functional and/or metabolic changes in lung cancer and/or response to therapeutic agents, such as kinase inhibitors.

Efforts have been made to determine protein signatures in lung cancer by using two-dimensional gel electrophoresis and subsequent protein identification by mass spectrometry assay or by using direct mass spectrometry analyses [5]. While this technology is useful for identification of proteins differentially expressed in tumor tissues, it is likely not adaptable to the rapid-throughput assays necessary for clinical application because of the time-intensive processes involved, the possibility of signal contaminations due to thousands of data points involved in the analysis, and the possible corruption of data sets due to experimental design issues [6].

The recent advent of protein microarray technology may allow us to identify critical nodes or interactions within the network of cellular signaling pathways. The advantage of the RPPA method is that a single test probe (antibody) is used for each array, so the testing condition is consistent for each antibody, thereby providing better reproducibility and sensitivity than other protein array techniques. With thoroughly assessed and validated antibodies, an RPPA can be used to detect signal differences in a few thousand molecules in testing samples [7]. Therefore, this technology is useful for monitoring changes in protein levels and protein phosphorylation over time, before and after treatment, between tumor and normal tissues, and between responders and non-responders. Once differential targets are identified, it is possible to use conventional methods to test a small subset of molecular biomarkers for prognosis or prediction of treatment response. To this end, we collected case-matched normal and malignant lung tissue samples of 101 patients and determined their protein levels and protein phosphorylation statuses using RPPA method and 126 antibodies. Here, we report that several molecular nodes that are critical in cell attachment, DNA repair, cell proliferation, and signal transduction were differentially expressed between normal and cancerous tissues, some of them were associated with clinical parameters, including survival outcomes.

## Results

### Patient and Tumor Characteristics

We collected case-matched normal and malignant lung tissue samples from 101 patients. Characteristics of those patients and the tumors are summarized in Table 1. The patients were ages 42–86 y, with a mean age of 65 y, and 55% were women. Most of the patients (93%) were Caucasian. Adenocarcinoma and squamous cell carcinoma accounted for 56% and 31% of histological types, respectively. The majority of patients (66%) had stage I disease. About 50% of tumors were poorly differentiated, and 40% were moderately differentiated. Ninety patients (89%) had a history of tobacco use/smoking. Twenty-four patients had neoadjuvant chemotherapy, and one had neoadjuvant radiotherapy.

**Table 1:** Clinical Information.

Pathology	Cases (numbers)	Ages (year)	Stage				Differentiation*			Sex#		Race§		
			I	II	III	IV	P	M	W	M	F	W	AA	His
Adenocarcinoma	57	44–86	36	9	11	1	27	24	6	23	34	53	2	2
Squamous Ca	31	42–82	20	6	5	0	17	13	1	19	12	29	1	1
Neuroendocrine	5	51–78	4	1	0	0	0	1	4	1	4	4	1	0
Large Cell/NSCLC	8	59–69	7	0	1	0	6	2	0	2	6	8	0	0
Total	101	65 (mean)	67	16	17	1	50	40	11	45	56	94	4	3

*P, M, W, stand for poorly, moderately and well differentiated, respectively.

# M and F stand for male and female, respectively.

§ W, AA and His stand for White, African American, and Hispanic, respectively.

### Differential Expression between Tumor and Normal Tissues Revealed by RPPA

**Figure 1:**
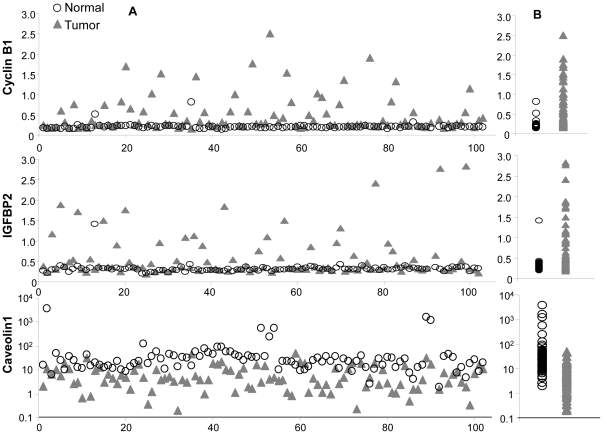
Signal intensity detected by RPPA. A) Signal intensity (Y axis) for each case (X axis) for molecules of cyclin B1, IGFBP2, and caveolin 1. B) Alighted distribution of signals in normal and tumor tissues.

For each sample and each antibody, the signal in the RPPA assays was compared between normal and tumor tissues. The signal difference between normal and tumor tissues was calculated as follows: [(mean of tumor tissues−mean of normal tissues)/(mean of normal tissues×100%)]. Of 126 proteins or phosphorylation sites analyzed, 18 had signal differences that were greater than 30% and were statistically significant (*p*<0.05) in all the normal and tumor samples analyzed (Table 2). These 18 molecules can be categorized as molecules associated with cell proliferation (cyclin B1), adaptor molecules in signal transduction (14-3-3zeta, IRS1-pS307, and IGFBP2), molecules in lipid metabolism (COX2 and ACC-pS79), molecules involved in DNA damage responses (Ku80, CHK2, and ATM), cell surface or matrix molecules (caveolin 1, CD31, and collagen type VI), and molecules in signaling pathways (PI3K/AKT pathway: PI3K-p85, mTOR, and S6K; Src/Stat pathway: Stat5 and Src; and MAP kinase pathway: p38-pT180). The signal intensities for cyclin B1, IGFBP2, and caveolin 1 in tissue samples from each case are shown as examples in Fig. 1. Most of these molecules have been reported to play critical roles in various cancers or to have altered expression in various cancers. For example, loss of caveolin 1 expression [8], [9] and overexpression of cyclin B1 [10], [11] in lung cancer tissue have previously been reported in studies with cDNA arrays and immunohistochemical analyses.

**Table 2:** Levels of proteins in 101 lung cancer cases.*

Molecules	Tumor	Normal	Difference	*P* value
Cyclin B1	0.49±0.03	0.21±0.03	134%	0.000
COX2	1.38±0.22	0.70±0.22	96%	0.032
14-3-3Zeta	3.94±0.33	2.15±0.33	84%	0.003
IGFBP2	0.58±0.04	0.33±0.04	79%	0.000
KU80	1.17±0.05	0.68±0.06	74%	0.000
CHK2	0.49±0.02	0.28±0.02	72%	0.000
ATM	1.63±0.04	0.97±0.04	69%	0.000
P38-pT180	0.63±0.02	0.39±0.02	63%	0.000
ACC-pS79	0.53±0.02	0.33±0.02	61%	0.000
IRS1-pS307	0.98±0.04	0.62±0.04	60%	0.000
STAT5	1.09±0.04	0.72±0.04	52%	0.000
S6	0.52±0.03	0.36±0.03	44%	0.006
SRC	3.60±0.05	2.66±0.05	35%	0.002
PI3K-p85	0.76±0.02	0.58±0.02	31%	0.000
mTOR	1.22±0.04	0.94±0.04	30%	0.000
Caveolin1	7.81±24.5	104.7±24.5	−93%	0.005
CD31	0.26±0.02	0.49±0.02	−48%	0.000
Collagen VI	1.32±0.09	2.27±0.09	−42%	0.000

* Values represent mean±SE.

Because 88 of the 101 cases in this study were either adenocarcinoma or squamous cell carcinoma, we analyzed whether these 18 molecules were significantly different between normal and tumor tissue samples for the two major subtypes of non-small cell lung cancers. The results showed that 13 of the 18 molecules were significantly different between normal and tumor tissue for both adenocarcinoma and squamous cell carcinoma (p≤0.05, Table S2), a finding similar to that observed when all samples were analyzed together. Two molecules (COX2 and S6) were not significantly different when adenocarcinoma and squamous cell carcinoma were analyzed separately, while PI3K-p85, Src, and mTOR remained significantly different between normal and tumor tissues in adenocarcinoma but not in squamous cell carcinoma. Whether the PI3K/mTOR/S6 and Src pathways are more critical in adenocarcinoma than in squamous cell carcinoma or whether this finding was due to smaller numbers of squamous cell carcinoma samples in the study is not clear.

### Validation of RPPA Data

**Figure 2:**
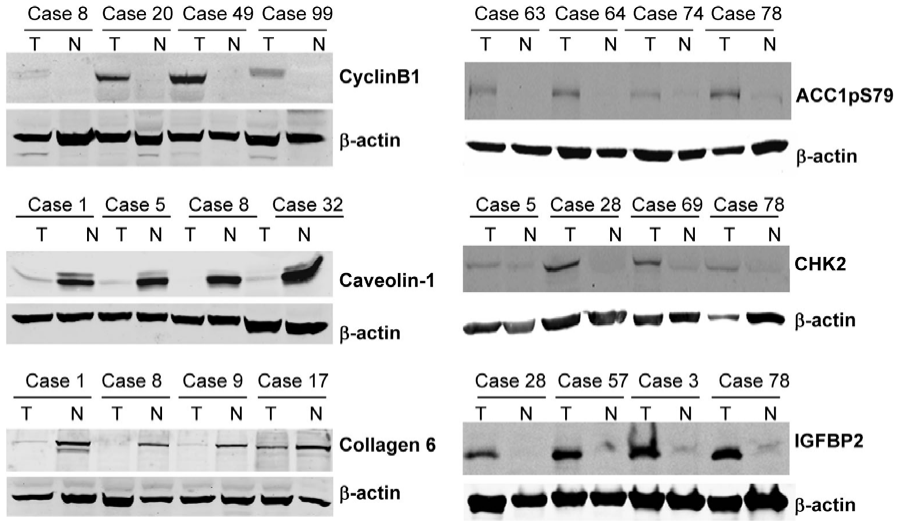
Protein levels detected by Western blot analysis. Cyclin B1, caveolin 1, collagen type VI, ACC-pS79, CHK2, and IGFBP2 in normal and primary lung tumor tissues were analyzed by Western blot in at least four cases in which RPPA showed signal difference in normal and tumor tissues. The Western blot results were consistent with those yielded by RPPA.

We performed Western blotting analysis on molecules whose expressions were changed in relatively a large number of tumor tissues, including cyclin B1, caveolin 1, collagen VI, ACC1/pS79, CHK2, and IGFBP2. The results showed that the data obtained from Western blot analysis matched those of RPPA assay (Fig. 2, Fig. S1), demonstrating that the data obtained from RPPA assay were reliable and can be validated by Western blot analysis.

**Figure 3:**
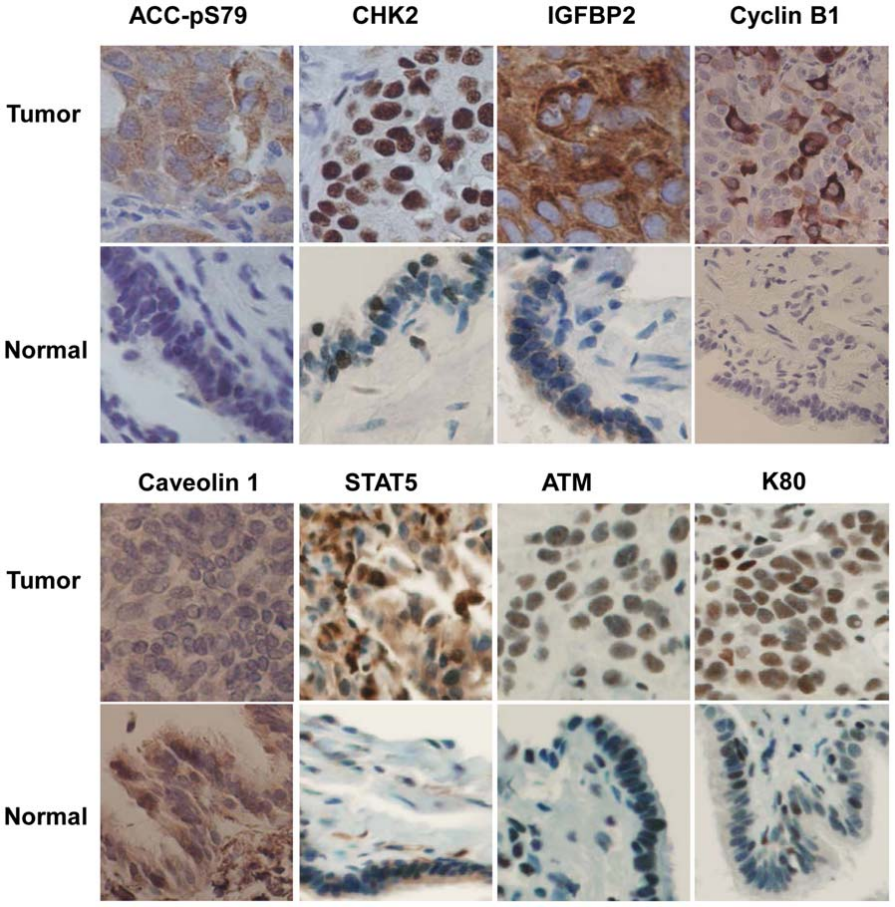
Examples of aberrant expression of 8 molecules in tumor tissue. Increased expression of ACC-pS79, CHK2, IGFBP2, cyclin B1, STAT5, ATM, and Ku80, and decreased expression of caveolin 1 in tumor tissues were compared with normal tissues from the same cases shown withconsistent with findings yielded by RPPA and Western blot analyses. 40× Magnification.

Neither RPPA assay nor Western blot analysis provided information about what types of cells, tumor or stromal, contributed to the differences observed. To determine the cell types in which the proteins were differentially expressed, we performed immunohistochemical analyses for five molecules (ACC-pS79, CHK2, IGFBP2, cyclin B1, and caveolin 1) on samples that showed difference between normal and tumor tissues. The results showed that the differences in the expression of all five molecules were derived from altered expression in cancer cells but not in stromal cells (Fig. 3). Striking heterogeneity in protein expression in tumor cells was observed for cyclin B1. Only a portion of tumor cells were stained strongly with cyclin B1 antibody whereas other tumor cells in the same tumor showed very low or negative staining for cyclin B1, possibly because of different status of cell cycles. Cyclin B1 expression is known to be cell cycle dependent and peaked at G2/M [12]. The overexpression or loss of expression of other molecules was much less heterogeneous.

Nanjundan *et al.* recently reported a RPPA profiling analysis on 46 lung cancer cases with 63 proteins or protein phosphorylation sites and identified several proteins were differentially expressed in primary lung cancer tissues [13]. We therefore compared the results of current study with that of Nanjundan's study. The two studies used completely separate sample sets. All samples used in Nanjundan's study were collected before 2000, while the samples used in this study were collected after 2006. Forty-eight proteins/protein phosphoryaltaion sites were tested in the both studies. Eight of eleven (72.7%) markers that were significantly different between normal and cancer tissues in Nanjundan's study have similar significant differences in the current studies. Three molecules (27.3%) (FAK, β-catenin and AKT) that were significantly different (p = 0.002–0.003) in Nanjundan's study were not significant in this study. This result indicates that validation of RPPA results from separate studies will be important, although the majority of the differently expressed molecules are consistent in the two studies.

Three (caveolin-1, cyclin B1 and Src-pY527) of four marker signature that differentiates NSCLC from normal lung in Nanjundan's study were also significantly different between normal and tumor tissues of the current studies. We therefore used Nanjundan's training set (25 cases) to test whether these three marker signature could be used to differentiate the whole data set (101 cases) of the current study. The result showed that these three markers, either alone or in combination, could distinguish tumor from the normal of the current study with various accuracies, sensitivities, and specificities (Table 3). In general, a combination of two or three markers improved either accuracy, sensitivity or specificity.

**Table 3:** Ability of using Nanjundan's training set to differentiate the whole data set of this study.

Molecules	Accuracy	Sensitivity	Specificity	PPV*	NPV*
Cyclin B1	0.755	0.539	0.971	0.948	0.678
Caveolin-1	0.794	0.745	0.843	0.826	0.768
SRC	0.775	0.735	0.814	0.798	0.755
Cyclin B/CAV-1	0.804	0.716	0.892	0.869	0.758
SRC/Cyclin B1	0.848	0.735	0.961	0.949	0.784
SRC/CAV-1	0.814	0.744	0.853	0.840	0.791
SRC/CyclinB1/CAV-1	0.828	0.765	0.892	0.876	0.791

PPV: Positive Predictive Value; NPV: Negative Predicative Value.

### Association with Clinical Data

**Figure 4:**
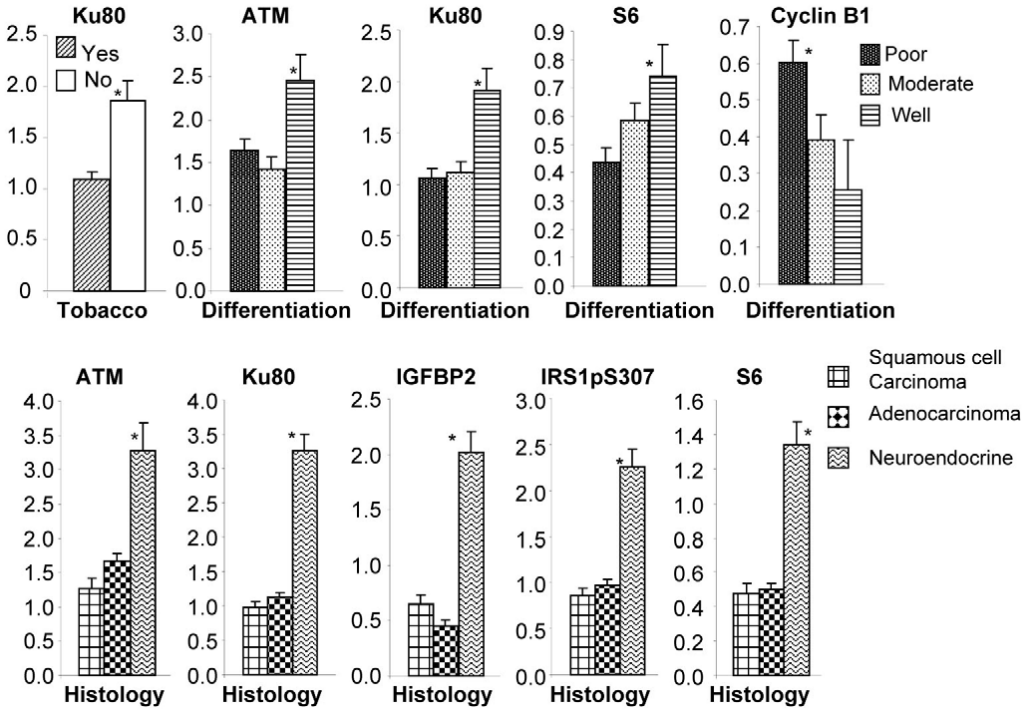
Protein levels in tumor tissues and association with clinical parameters. Protein levels in tumor tissues detected in RPPA assay were analyzed for associations with clinical parameters of patients. The molecule that was significantly different in tumors based on clinical parameters analyzed is shown on the top of each graph. The clinical parameters are shown at the bottom of each graph. The histology and differentiation diagnoses were based on pathological reports in clinical database. * indicates that the difference was significant when compared with other groups in the same graph (*p*<0.05).

We analyzed whether expression of the 18 molecules listed in Table 2 in tumor tissues was associated with any clinical parameters. Statistic analysis revealed that levels of these molecules in tumor tissues were not significantly associated with clinical stage or gender. However, expression of Ku80 was significantly higher in the samples of patients without smoking history than those with smoking history (*p* = 0.004). Expression of cyclin B1 was significantly higher in poorly differentiated tumor tissues than in moderately or well-differentiated tumor tissues (*p*<0.025). On the other hand, expression of ATM, Ku80, and S6 was significantly higher in well-differentiated tumor tissues than in poorly or moderately differentiated tumor tissues (Fig. 4). When expression in different histological types was compared, the expressions of ATM, Ku80, IGFBP2, IRS1-pS307, and S6 were significantly higher in neuroendocrinal carcinoma than in adenocarcinoma or squamous cell carcinoma (*p*<0.05). This result suggests that expression of certain molecules were dramatically different in neuroendocrinal tumors when compared with those in adenocarcinoma or squamous cell cancer, whereas the levels of the differentially expressed proteins listed in Table 1 were more or less similar between adenocarcinoma and squamous cell cancer. Nevertheless, because of relatively low numbers of neuroendocrinal tumors used in this study, it is not clear whether there exists a specific molecular signature for this type of cancer.

### Association with Survival Outcomes

**Figure 5:**
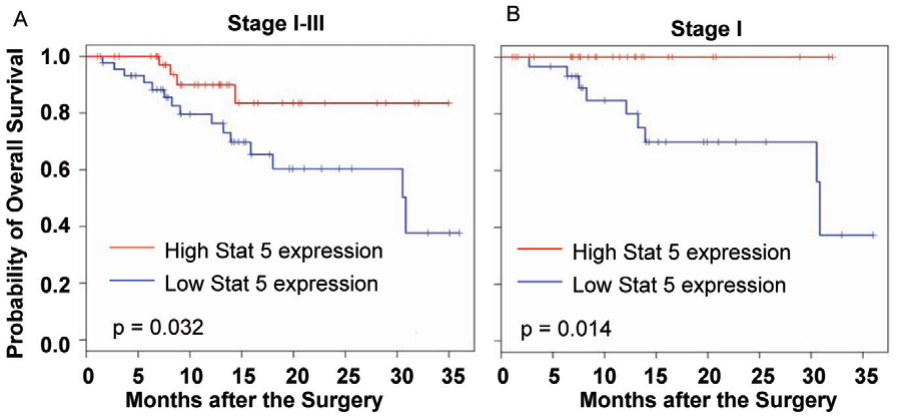
Association with Survival Outcomes. Kaplan-Meier analysis on association of Stat5 levels and survival outcomes for Stage I–III (A) (n = 50 for each group), and Stage I only (B) patients (n = 33 for STAT5 high group, 34 for STAT5 low group).

To determine whether levels of those proteins are associated with clinical outcomes, we performed survival analysis on the differentially expressed protein markers shown in Table 1, using Kaplan-Meier method. Briefly, we separate the patients into two groups based on the median expression value of each individual marker, designated as high and low expression groups, and then using the Kaplan-Meier algorithm to compute survival curves for the two defined groups of each marker. The result showed that the levels of Stat5 were significantly associated with survival outcomes when analyzed with both stage I–III patients (p = 0.032) and with stage I patients only (p = 0.014) (Fig. 5). Patients with a high level of Stat5 in their tumor tissues had favorable survival outcomes when compared with those with a lower level of Stat5, suggesting that Stat5 could be a useful marker for prognosis of lung cancers.

## Discussion

We analyzed molecular differences in protein or protein phosphorylation levels between normal and lung cancer tissues in 101 samples by RPPA assay. Of 126 molecules analyzed, we identified 18 molecules that were dramatically (>30%) and statistically significant (*p*<0.05) different between normal and tumor samples. Western blot analysis and/or immunohistopathologic assays of several molecules validated the results obtained from RPPA arrays, demonstrating that the results from RPPA analysis are reliable. Moreover, a comparison with a RPPA study performed on another of patient samples showed that the results of RPPA profiling were highly repeatable and consistent in separate studies with separate set of patient samples. Our results also indicate that the expression of several molecules in tumor tissues was associated with smoking history, differentiation, and histopathologic types of lung cancers.

A number of biomarkers identified here are consistent with those reported in the literature in terms of their altered gene expressions in tumor tissues, including caveolin 1 [8], [9], cyclin B1 [10], [11], 14-3-3zeta [14], Stat5 [15], activated p38 [16], and IGFBP2 [17]. However, for some molecules, some contradictory findings were reported by others previously. For example, CHK2 expression or its activation was found to be diminished in non-small-cell lung cancer tumor tissues of a commercially available tissue array [18], or increased in 50% of surgically resected lung and breast tumor specimens from untreated patients [19]. We found that CHK2 expression was increased in both adenocarcima and squamous cell carcinoma lung tissues, consisting with that reported by DiTullio et al [19]. Increased COX2 expression was found in our study, but it was less frequent than reported by Hida et. al in Japanese patients, where a significant increase in COX2 expression was observed in 70% of invasive adenocarcinoma cases [20]. Our result was consistent with that reported by Khuri et al, who observed that only a few cases had strong COX2 expression in tumor tissues [21].

Interestingly, our results showed that several molecules involved in DNA damage/repair (ATM, CHK2 and Ku80) were increased in tumor tissues. Increased mRNA levels of ATM and DNA-PKcs, but not of Ku80, were detected in tumor tissues when compared with adjacent normal tissues [22]. However, little is known about ATM protein expression in primary lung cancer tissues. ATM, CHK2, and/or Ku80 are regarded as tumor suppressor genes that are involved in DNA damage response [23], [24]. Interestingly, constitutive activation of ATM/CHK2 pathway was found in p53 mutant cancer cells [19]. The increase of those DNA damage response/repairing molecules in tumor tissues may reflect the presence of genome instability in cancer cells, a common feature that distinguish cancers from normal tissues [25]. It is noteworthy that overexpression of Ku80 was found in head and neck cancer and in skin cancer [26], [27], and could be caused by activation of NFκB and COX2 [28]. Alternatively, increased expression of Ku80 in non-smoker patients or neuroendocrine tumors may reflect a high demand for repair of double strand breaks by nonhomologous end-joining in those cancer tissues because Ku80 is critical in this DNA repair pathway. Those molecules may serve as a marker for cancer therapy targeting the DNA repair pathway [29]. Because twenty-five patients included in this study had various neoadjuvant chemotherapies or radiotherapy, we analyzed whether increased expression of those molecules was associated with chemo- and radiotherapy. Statistical analysis showed that increased expression of ATM, CHK2, and Ku80 was not associated with neoadjuvent chemotherapy or radiotherapy. Thus, the increased expression of these DNA damage/repair molecules was unlikely induced by treatment but rather an intrinsic characteristic of primary tumors.

Several deregulated proteins identified here have been investigated as therapeutic targets for cancer therapy. Small molecules or kinase inhibitors targeting growth factor receptors and the PI3K/AKT/mTOR, Src/Stat, p38, and ATM/CHK2 pathways were extensively investigated for cancer treatment, both preclinically and clinically, including treatment for lung cancers [29]–[31]. A recent study showed that inhibition of ATM or CHK2 is sufficient to sensitize p53-deficient tumor cells, but not p53 wild-type cells, to genotoxic chemotherapeutic agent cisplatin or doxorubicin [32], suggesting that combination of cisplatin or doxorubin with ATM or CHK2 inhibitors could benefit patients carrying p53 mutant tumors. Our results also showed that increased expression of STAT5 may serve as favorable prognostic biomarker for lung cancer patients treated with surgery. Although the underlying mechanisms remain to be further investigated, STAT5 as a tumor marker of favorable prognosis has been reported for breast cancer [33]–[35] and nasopharyngeal cancer [36]. Evidence showed that STAT5 promotes homotypic adhesion and inhibits invasive characteristics of human breast cancer cells [34]. Whether the same occurs to lung cancer cells remain to be further investigated. However, the significant association of STAT5 with clinical outcomes, in particular in stage I lung cancer, suggested that STAT5 might be a useful prognostic biomarker for lung cancer.

## Materials and Methods

### Human Lung Tissue Specimens

Normal and malignant lung tissue samples were collected between 2006 and 2009 from surgically removed specimens under a research protocol Lab-90-020 with informed consent from the patients. The study was approved by local ethics committee (the Institutional Review Board at The University of Texas MD Anderson Cancer Center). The normal tissues were at least 5 cm away from the edge of corresponding tumors in the same specimens. Both normal and tumor tissues were collected from the operating room immediately after specimens were removed from patients. In all cases, histology quality control was performed by a thoracic pathologist on tissue sections. Tumor samples were included in the analysis if the percentage of malignant cells present in the sample were ≥70%. Normal lung samples from the same patients were reviewed to confirm that they contained no malignant cells. All samples were divided into two portions: one portion was instantly frozen and stored in liquid nitrogen for protein extraction; the other portion was fixed in formalin and embedded in paraffin for routine histological or immunohistochemical examinations. The pairs of matched samples were harvested, processed, and analyzed at the same time, under the same protocols.

### RPPA Assay

RPPA assay was performed at the Functional Proteomics Reverse Phase Protein Array Core facility at our institution as we previously described [37]. Briefly, the tissue samples were washed twice in ice-cold PBS and then homogenized in RPPA lysis buffer [1% Triton X-100, 50 mmol/L HEPES (pH 7.4), 150 mmol/L NaCl, 1.5 mmol/L MgCl_2_, 1 mmol/L EGTA, 100 mmol/L NaF, 10 mmol/L NaPPi, 10% glycerol, 1 mmol/L Na_3_VO_4_, 1 mmol/L phenylmethylsulfonyl fluoride, and 10 µg/mL aprotinin]. After centrifugation, the supernatant was collected, and the protein concentration was determined by routine (*e.g.*, Bradford) assays and then adjusted to 1–1.5 mg/ml by addition lysis buffer. The tissue lysates were mixed with 1/4 volume of 4× SDS sample buffer containing 40% glycerol, 8% SDS, 0.25 M Tris-HCl (pH 6.8), and 10% (v/v) 2-mercaptoethanol (freshly added). Two-fold serially diluted tissue lysates (from undiluted to 1∶16 dilution) were printed on nitrocellulose-coated slides (Whatman, Inc.) by using a GeneTAC G3 arrayer (Genomic Solutions), along with corresponding positive and negative controls prepared from the dilution buffer. A total of 126 validated antibodies specific for proteins or their phosphorylated sites that are involved in various signaling pathways were available and used in the RPPA (see Table S1 for antibodies used in this study). Each slide was probed with a validated primary antibody plus a biotin-conjugated secondary antibody. The signal was amplified using a DakoCytomation catalyzed system (Dako) and visualized by 3,3′-diaminobenzidine tetrahydrochloride colorimetric reaction. The slides were scanned, analyzed, and quantified using customized software, Microvigene (VigeneTech, Inc.), to generate spot intensity. Signals from each dilution were fitted with the non-parametric model developed by the Department of Bioinformatics and Computational Biology at MD Anderson [38]. The protein concentrations of each set of slides were then normalized and corrected across samples by the linear expression values, using the median expression levels of all antibody experiments to calculate a loading correction factor for each sample, as previously described [13], [37].

### Western Blot Analysis

To validate the results from RPPA assays, we performed Western blot analysis for a subset of molecules that showed significant difference between normal and cancer tissues. About 40 mg of each frozen tissue sample was washed twice in cold PBS and homogenized in 0.5 ml ice-cold lysis buffer. Extracts equivalent to 50–60 µg of the total protein were separated by 10% SDS-polyacrylamide gel electrophoresis, then transferred to nitrocellulose membranes. The Western blot analysis was performed as previously described [37]. Antibodies for IGFBP2, caveolin-1, CHK2 (1C12), and phospho-acetyl-CoA carboxylase (ACC-pS79) were purchased from Cell Signaling, antibody for cyclin B1 was from Epitomics, and collagen type VI from Santa Cruz Biotechnology.

### Immunohistochemical Staining and Evaluation

The same antibodies used for Western blot analysis were used for immunohistochemical staining. Formalin-fixed and paraffin-embedded tissue sections (5-µm thick) were deparaffinized, hydrated, and heated in a steamer for antigen retrieval. The slides were then stained with various antibodies as described above. Tissues not incubated with a control antibody to mice IgG instead of a primary antibody were used as a negative control.

### Statistical Analysis

Analysis of variance was performed by using STATISTICA software (StatSoft, Inc.) for comparisons among groups. Student's *t* test was used for comparison between two groups. The diagonal linear discriminant analysis (DLDA) was used for classification and prediction of normal and tumor tissues. The survival data will be analyzed using the Kaplan-Meier method and Cox's proportional model. A *p*-value of <0.05 was considered statistically significant.

## Supporting Information

Figure S1 **Protein levels detected by Western blot analysis in 6 additional cases for IGFBP2 and CHK2.** IGFBP2 and CHK2 in normal (N) and primary lung tumor (T) tissues were analyzed by Western blot in additional 6 cases in which RPPA showed signal difference in normal and tumor tissues. β-actin was used as loading control.
(TIF)

Table S1 Expression difference in adenocarcinoma and squamous cancer.*
(DOC)

Table S2 **Proteins and phosphorylation sites used in RPPA studies.**
(DOC)

## References

[1] Bhattacharjee A, Richards WG, Staunton J, Li C, Monti S (2001). Classification of human lung carcinomas by mRNA expression profiling reveals distinct adenocarcinoma subclasses.. Proc Natl Acad Sci USA.

[2] Volinia S, Calin GA, Liu CG, Ambs S, Cimmino A (2006). A microRNA expression signature of human solid tumors defines cancer gene targets.. Proc Natl Acad Sci USA.

[3] Hung RJ, McKay JD, Gaborieau V, Boffetta P, Hashibe M (2008). A susceptibility locus for lung cancer maps to nicotinic acetylcholine receptor subunit genes on 15q25.. Nature.

[4] Nishizuka S, Charboneau L, Young L, Major S, Reinhold WC (2003). Proteomic profiling of the NCI-60 cancer cell lines using new high-density reverse-phase lysate microarrays.. Proc Natl Acad Sci USA.

[5] Yanagisawa K, Tomida S, Shimada Y, Yatabe Y, Mitsudomi T (2007). A 25-signal proteomic signature and outcome for patients with resected non-small-cell lung cancer.. J Natl Cancer Inst.

[6] Hu J, Coombes KR, Morris JS, Baggerly KA (2005). The importance of experimental design in proteomic mass spectrometry experiments: some cautionary tales.. Briefings in Functional Genomics & Proteomics.

[7] Paweletz CP, Charboneau L, Bichsel VE, Simone NL, Chen T (2001). Reverse phase protein microarrays which capture disease progression show activation of pro-survival pathways at the cancer invasion front.. Oncogene.

[8] Wikman H, Kettunen E, Seppanen JK, Karjalainen A, Hollmen J (2002). Identification of differentially expressed genes in pulmonary adenocarcinoma by using cDNA array.. Oncogene.

[9] Wikman H, Seppanen JK, Sarhadi VK, Kettunen E, Salmenkivi K (2004). Caveolins as tumour markers in lung cancer detected by combined use of cDNA and tissue microarrays.. J Pathol.

[10] Amatschek S, Koenig U, Auer H, Steinlein P, Pacher M (2004). Tissue-wide expression profiling using cDNA subtraction and microarrays to identify tumor-specific genes.. Cancer Res.

[11] Soria JC, Jang SJ, Khuri FR, Hassan K, Liu D (2000). Overexpression of cyclin B1 in early-stage non-small cell lung cancer and its clinical implication.. Cancer Res.

[12] Pines J, Hunter T (1989). Isolation of a human cyclin cDNA: evidence for cyclin mRNA and protein regulation in the cell cycle and for interaction with p34cdc2.. Cell.

[13] Nanjundan M, Byers LA, Carey MS, Siwak DR, Raso MG (2010). Proteomic profiling identifies pathways dysregulated in non-small cell lung cancer and an inverse association of AMPK and adhesion pathways with recurrence.. J Thor Oncol.

[14] Fan T, Li R, Todd NW, Qiu Q, Fang HB (2007). Up-regulation of 14-3-3zeta in lung cancer and its implication as prognostic and therapeutic target.. Cancer Res.

[15] Sanchez-Ceja SG, Reyes-Maldonado E, Vazquez-Manriquez ME, Lopez-Luna JJ, Belmont A (2006). Differential expression of STAT5 and Bcl-xL, and high expression of Neu and STAT3 in non-small-cell lung carcinoma.. Lung Cancer.

[16] Greenberg AK, Basu S, Hu J, Yie TA, Tchou-Wong KM (2002). Selective p38 activation in human non-small cell lung cancer.. Am J Resp Cell Mol Biol.

[17] Migita T, Narita T, Asaka R, Miyagi E, Nagano H (2010). Role of insulin-like growth factor binding protein 2 in lung adenocarcinoma: IGF-independent antiapoptotic effect via caspase-3.. Am J Pathol.

[18] Zhang P, Wang J, Gao W, Yuan BZ, Rogers J (2004). CHK2 kinase expression is down-regulated due to promoter methylation in non-small cell lung cancer.. Molecular Cancer.

[19] DiTullio RA, Mochan TA, Venere M, Bartkova J, Sehested M (2002). 53BP1 functions in an ATM-dependent checkpoint pathway that is constitutively activated in human cancer.[Erratum appears in Nat Cell Biol. 2003 Jan;5(1):84.].. Nature Cell Biology.

[20] Hida T, Yatabe Y, Achiwa H, Muramatsu H, Kozaki K (1998). Increased expression of cyclooxygenase 2 occurs frequently in human lung cancers, specifically in adenocarcinomas.. Cancer Res.

[21] Khuri FR, Wu H, Lee JJ, Kemp BL, Lotan R (2001). Cyclooxygenase-2 overexpression is a marker of poor prognosis in stage I non-small cell lung cancer.. Clinical Cancer Res.

[22] Xing J, Wu X, Vaporciyan AA, Spitz MR, Gu J (2008). Prognostic significance of ataxia-telangiectasia mutated, DNA-dependent protein kinase catalytic subunit, and Ku heterodimeric regulatory complex 86-kD subunit expression in patients with nonsmall cell lung cancer.. Cancer.

[23] Matsuoka S, Ballif BA, Smogorzewska A, McDonald ER, Hurov KE (2007). ATM and ATR substrate analysis reveals extensive protein networks responsive to DNA damage.. Science.

[24] Hirao A, Kong YY, Matsuoka S, Wakeham A, Ruland J (2000). DNA damage-induced activation of p53 by the checkpoint kinase Chk2.. Science.

[25] Halazonetis TD, Gorgoulis VG, Bartek J (2008). An oncogene-induced DNA damage model for cancer development.. Science.

[26] Moeller BJ, Yordy JS, Williams MD, Giri U, Raju U (2011). DNA repair biomarker profiling of head and neck cancer: Ku80 expression predicts locoregional failure and death following radiotherapy.. Clinical Cancer Research.

[27] Parrella P, Mazzarelli P, Signori E, Perrone G, Marangi GF (2006). Expression and heterodimer-binding activity of Ku70 and Ku80 in human non-melanoma skin cancer.. Journal of Clinical Pathology.

[28] Lim JW, Kim H, Kim KH (2002). Expression of Ku70 and Ku80 mediated by NF-kappa B and cyclooxygenase-2 is related to proliferation of human gastric cancer cells.. J Biol Chem.

[29] Bolderson E, Richard DJ, Zhou BB, Khanna KK (2009). Recent advances in cancer therapy targeting proteins involved in DNA double-strand break repair.. Clinical Cancer Reearch.

[30] Janne PA, Gray N, Settleman J (2009). Factors underlying sensitivity of cancers to small-molecule kinase inhibitors.. Nature Rev Drug Discovery.

[31] Noble ME, Endicott JA, Johnson LN, Noble MEM, Endicott JA (2004). Protein kinase inhibitors: insights into drug design from structure.. Science.

[32] Jiang H, Reinhardt HC, Bartkova J, Tommiska J, Blomqvist C (2009). The combined status of ATM and p53 link tumor development with therapeutic response.. Gene Dev.

[33] Nevalainen MT, Xie J, Torhorst J, Bubendorf L, Haas P (2004). Signal transducer and activator of transcription-5 activation and breast cancer prognosis.. J Clin Oncol.

[34] Sultan AS, Xie J, LeBaron MJ, Ealley EL, Nevalainen MT, Rui H (2005). Stat5 promotes homotypic adhesion and inhibits invasive characteristics of human breast cancer cells.. Oncogene.

[35] Yamashita H, Nishio M, Ando Y, Zhang Z, Hamaguchi M (2006). Stat5 expression predicts response to endocrine therapy and improves survival in estrogen receptor-positive breast cancer.. Endocrine-Related Cancer.

[36] Hsiao JR, Jin YT, Tsai ST, Shiau AL, Wu CL (2003). Constitutive activation of STAT3 and STAT5 is present in the majority of nasopharyngeal carcinoma and correlates with better prognosis.. Br J Cancer.

[37] Wei X, Guo W, Wu S, Wang L, Lu Y (2009). Inhibiting JNK dephosphorylation and induction of apoptosis by novel anticancer agent NSC-741909 in cancer cells.. J Biol Chem.

[38] Hu J, He X, Baggerly KA, Coombes KR, Hennessy BT (2007). Non-parametric quantification of protein lysate arrays.. Bioinformatics.

