# Supplementary figures and images for: Aberrant Expression of Proteins Involved in Signal Transduction and DNA Repair Pathways in Lung Cancer and Their Association with Clinical Parameters
Source: PLoS One. 2012 Feb 10;7(2):e31087. doi: 10.1371/journal.pone.0031087 (PMC3277494; doi:10.1371/journal.pone.0031087)

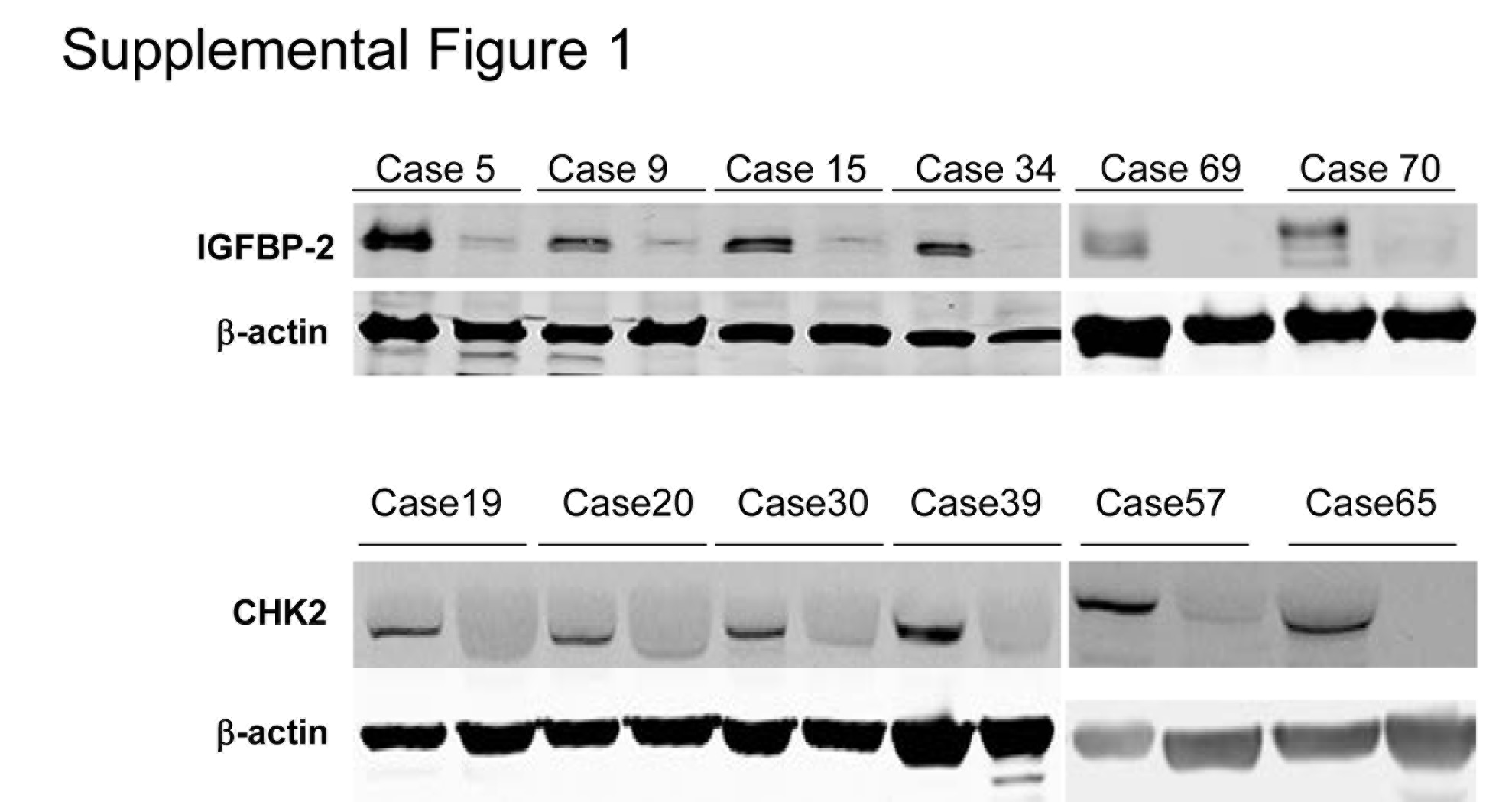

Supplement: Figure S1 — Protein levels detected by Western blot analysis in 6 additional cases for IGFBP2 and CHK2. IGFBP2 and CHK2 in normal (N) and primary lung tumor (T) tissues were analyzed by Western blot in additional 6 cases in which RPPA showed signal difference in normal and tumor tissues. β-actin was used as loading control. (TIF) [file pone.0031087.s001.tif]
