# Supplementary material for: Aberrant Expression of Proteins Involved in Signal Transduction and DNA Repair Pathways in Lung Cancer and Their Association with Clinical Parameters
Source: PLoS One. 2012 Feb 10;7(2):e31087. doi: 10.1371/journal.pone.0031087 (PMC3277494; doi:10.1371/journal.pone.0031087)
Supplement: Table S1 — Expression difference in adenocarcinoma and squamous cancer.* (DOC) [file pone.0031087.s002.doc]

**Supplemental Table 1.** Expression difference in adenocarcinoma and squamous cancer*

* Values represent mean±SE

| **Molecules** | **Adenocarcinoma** | | | | **Squamous Carcinoma** | | | |
| --- | --- | --- | --- | --- | --- | --- | --- | --- |
|  | Tumor | Normal | Difference | *P* value | Tumor | Normal | Difference | *P* value |
| Cyclin B1 | 0.44±.03 | 0.20±.03 | 115% | 0.000 | 0.53±.06 | 0.22±.06 | 139% | 0.005 |
| ACC-pS79 | 0.55±.02 | 0.33±.02 | 67% | 0.000 | 0.48±.03 | 0.35±.03 | 35% | 0.016 |
| KU80 | 1.12±.05 | 0.68±.05 | 66% | 0.000 | 1.11±.09 | 0.70±.09 | 57% | 0.030 |
| ATM | 1.66±.10 | 1.00±.10 | 66% | 0.000 | 1.47±.13 | 1.00±.13 | 47% | 0.050 |
| CHK2 | 0.46±.02 | 0.28±.02 | 65% | 0.000 | 0.51±.04 | 0.29±.04 | 76% | 0.001 |
| P38-pT180 | 0.65±.03 | 0.39±.03 | 64% | 0.000 | 0.59±.04 | 0.39±.04 | 53% | 0.002 |
| STAT5 | 1.21±.06 | 0.64±.06 | 64% | 0.000 | 0.90±.05 | 0.69±.05 | 23% | 0.050 |
| IRS1-pS307 | 0.97±.05 | 0.63±.05 | 54% | 0.000 | 0.89±.07 | 0.62±.07 | 43% | 0.050 |
| 14-3-3Zeta | 3.09±.25 | 2.03±.25 | 52% | 0.031 | 5.20±.86 | 2.33±.86 | 123% | 0.020 |
| PI3K-p85 | 0.80±.03 | 0.57±.03 | 40% | 0.000 | 0.69±.04 | 0.62±.04 | 12% | 0.650 |
| SRC | 3.80±.22 | 2.76±.22 | 38% | 0.013 | 3.19±.20 | 2.68±.20 | 19% | 0.394 |
| mTOR | 1.31±.05 | 0.95±.05 | 37% | 0.000 | 1.04±.05 | 0.96±.05 | 8% | 0.755 |
| IGFBP2 | 0.45±.03 | 0.33±.03 | 37% | 0.005 | 0.69±.09 | 0.32±.09 | 116% | 0.046 |
| COX2 | 1.06±.17 | 0.69±.17 | 35% | 0.125 | 0.88±.06 | 0.71±.06 | 19% | 0.226 |
| S6 | 0.50±.04 | 0.37±.04 | 26% | 0.171 | 0.49±.04 | 0.35±.04 | 29% | 0.158 |
| Caveolin1 | 8.86±40.9 | 133.0±40.9 | -93% | 0.033 | 7.36±22.1 | 81.5±22.1 | -91% | 0.020 |
| Collagen VI | 1.27±.11 | 2.24±.11 | -43% | 0.000 | 1.44±.17 | 2.39±.17 | -40% | 0.003 |
| CD31 | 0.28±.03 | 0.49±.03 | -42% | 0.000 | 0.23±.04 | 0.51±.04 | -54% | 0.000 |
