# Supplementary material for: Aberrant Expression of Proteins Involved in Signal Transduction and DNA Repair Pathways in Lung Cancer and Their Association with Clinical Parameters
Source: PLoS One. 2012 Feb 10;7(2):e31087. doi: 10.1371/journal.pone.0031087 (PMC3277494; doi:10.1371/journal.pone.0031087)
Supplement: Table S2 — Proteins and phosphorylation sites used in RPPA studies. (DOC) [file pone.0031087.s003.doc]

| 14-3-3Beta  **Supplemental Table 2**. Proteins and phosphorylation sites used in RPPA studies | cMyc/pT58 | GATA3 | p38 | S6 |
| --- | --- | --- | --- | --- |
| 14-3-3Zeta | cMyc | GSK3ab/pT21 | p53 | SMAD3/pS423 |
| 4EBP1/pS65 | cRAF | GSK3ab | p70S6K/pT389 | SMAD3 |
| 4EBP1/pT37 | Caspase3 | HER2/pY1248 | p70S6K | SRC/pY416 |
| 4EBP1/pT70 | Caspase7 | HER2 | p85/PI3K | SRC/pY527 |
| 4EBP1 | Caveolin-1 | HSP27 | p85/PI3Ka | SRC |
| ACC/pS79 | CD31 | IGFBP2 | p90RSK/pT359 | STAT3/pT727 |
| AIB1 | CHK1/pS345 | IGFR1/pY1135 | p90RSK | STAT3/pY705 |
| AKT/pS473 | CHK1 | IRS1/pS307 | PARP/cleaved | STAT3 |
| AKT/pT308 | CHK2/pT68 | IRS1 | PARP | STAT5/pY694 |
| AKT | CHK2 | JNK2 | PAX2 | STAT5 |
| AMPKA/pT272 | Collagen VI | KU80 | PCNA | STAT6/pY641 |
| AMPKA | COX2 | LKB1 | PDK1/pS241 | Stathmin |
| AR | Cyclin B1 | MAPK/pT202 | PDK1 | TAU |
| ATM/pS1981 | Cyclin E1 | MEK1,2/pS217 | PI3K/p110a | TAZ |
| ATM | Ecadherin | MEK1 | PKCa/pS657 | Telomerase |
| ATR/pS428 | EGFR/pY1173 | MGMT | PKCa | TSC2/pT1462 |
| ATR | EGFR/pY992 | MRE11 | PR | TSC2 |
| ATRIP | EGFR | MSH2 | PTCH | VASP |
| BetaCatenin | elF4E | mTOR | PTEN | VEGFR2 |
| BCl2 | ER/pS118 | Ncadherin | Rab25 | XIAP |
| BIM | ERK2 | NCKIPS1 | RAD51 | XRCC1 |
| BRCA2 | ETV6 | Notch3 | Rb/pS807 | YAP |
| cJUN/pS73 | FAK | p21 | Rb |  |
| cJUN | FKHRL/FOXO3 | p27 | S6/pS235 |  |
| cKIT | FOXO3a/pS318 | p38/pT180 | S6/pS240 |  |
